# eIF3i activity is critical for endothelial cells in tumor induced angiogenesis through regulating VEGFR and ERK translation

**DOI:** 10.18632/oncotarget.15274

**Published:** 2017-02-11

**Authors:** Yaguang Zhang, Ping Wang, Qian Zhang, Xiaomin Yao, Linjie Zhao, Yibin Liu, Xiaowei Liu, Rui Tao, Chuan Yu, Yuhao Li, Xiangrong Song, Shaohua Yao

**Affiliations:** ^1^ State Key Laboratory of Biotherapy/Collaborative Innovation Center of Biotherapy, West China Hospital, Sichuan University, Chengdu, China; ^2^ Key Laboratory of Tumor Microenvironment and Neurovascular Regulation, Nankai University School of Medicine, Tianjin, China

**Keywords:** eIF3i, selective translational control, tumor angiogenesis, VEGFR, ERK

## Abstract

Translational control is a critical step in the regulation of gene expression. Accumulating evidence shows that translational control of a subgroup of mRNAs tends to be selective. However, our understanding of the function of selective translational control in endothelial cells is still incomplete. We found that a key translational regulator, eIF3i, is highly expressed in endothelial cells during embryonic and tumor angiogenesis. Knockdown of eIF3i restrained cell proliferation and migration in endothelial cells. In zebrafish angiogenesis model, eIF3i mutant endothelial cells could not respond to induction signals from tumor mass. Mechanistically, we showed that eIF3i knockdown reduced VEGFR/ERK signaling by down-regulating VEGFR2 and ERK protein expression. Gene therapy model suggested that the growth and metastasis of cancer cells were suppressed by eIF3i shRNA. Therefore, our work established a selective translational regulatory mechanism during tumor induced angiogenesis and suggested that targeting eIF3i may be applicable for anticancer therapy.

## INTRODUCTION

Translational control is one of the most determining steps for final gene expression, deregulation of which causes abnormal gene expression, leading to altered cell growth and possibly oncogenesis [1–5]. Accumulating evidence shows that the translation of a subgroup of mRNAs tends to be selectively controlled, which enable cells to make quick response. In endothelial cells, selective translational control is significantly utilized when endothelial cells respond to various stimuli from environment [6]. During hypoxia, global translation is inhibited, but a subset of mRNAs could be selectively translated. For example, translation rate of an endothelium-specific receptor tyrosine kinase for angiopoietins (Tie2) is significantly increased under prolonged and severe hypoxia [7]. In addition, fluid shear stress can also stimulate translational activity in vascular endothelial cells. For instance, fluid forces can rapidly activate the translation of pp70^S6k^ mRNA to modify endothelial phenotype [8]. However, it is still unknown whether selective translational control also functions in tumor induced angiogenesis.

eIF3i is one out of 13 subunits of the mammalian eIF3 complex which associated with the 40S ribosome and facilitates the formation of 43S pre-initiation complex [9]. Overexpression of eIF3i has been found in several types of human cancers, including human colon adenocarcinoma and adenoma, human hepatocelluar carcinoma, head and neck squamous cell carcinomas, breast cancer, cervical cancer and metastatic melanoma [10–14]. eIF3i is shown to act as a signaling transducer by bind to and activating AKT1 through preventing PP2A-induced AKT1 dephosphorylation in tumor cells [14]. In addition, eIF3i could promote tumor angiogenesis through up-regulating VEGFA translation [15].

Here, we show that eIF3i is critical for selective translational control in endothelial cells during tumor angiogenesis. We show that eIF3i is highly expressed in zebrafish endothelial cells during embryonic angiogenesis and in human umbilical vein endothelial cells (HUVECs) activated by tumor cell derived signals. Silence of eIF3i restrains cell proliferation and migration in HUVECs, which are accompanied by a significant reduction in the phosphorylation of ERK but not AKT. Mechanistically, we demonstrate that eIF3i selectively control the translation of ERK and VEGFR2. Inhibition of eIF3i with liposome delivered eIF3i shRNA efficiently reduces tumor metastasis.

## RESULTS

### eIF3i is highly expressed in endothelial cell during embryonic and tumor angiogenesis

**Figure 1:**
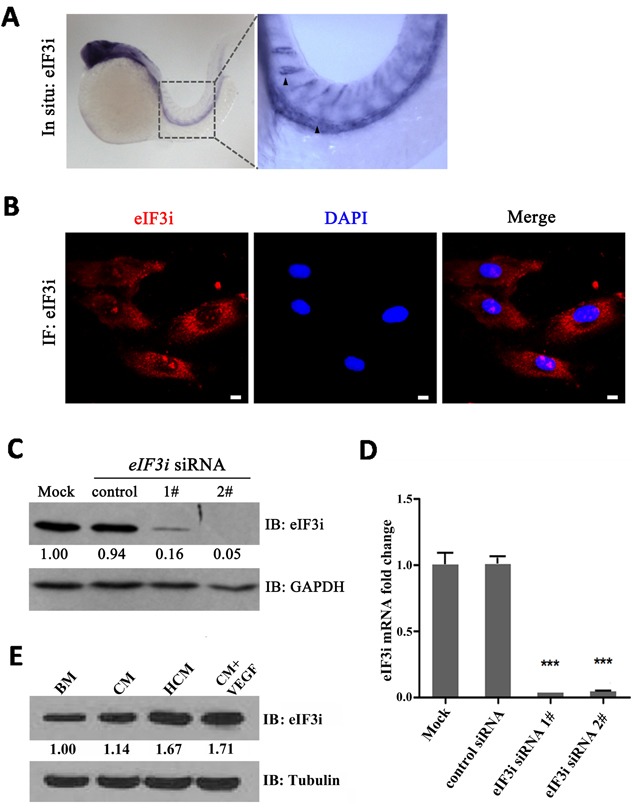
eIF3i is expressed in zebrafish and mammalian endothelial cells **A**. eIF3i is expressed in endothelial cells of 24 hpf zebrafish embryos as determined by WISH. The black arrowheads point to the dorsal aorta and intersegment vessels of embryos. **B**. Human umbilical vein endothelial cells (HUVECs) expressed eIF3i. The red channel represents eIF3i, and the blue channel represents the cell nucleus. Scale bars, 10μm. **C**. eIF3i siRNAs significantly inhibited eIF3i protein level. GAPDH was used as a loading control. Fold changes are compared with mock and presented below each blot. **D**. eIF3i siRNAs significantly inhibited eIF3i mRNA level. (n=3).***p<0.001. **E**. HCM increased the expression of eIF3i in HUVECs. Tubulin was used as a loading control. Fold changes are compared with BM and presented below each blot. BM, basic medium; CM, B16 cell non-hypoxic conditional medium; HCM, B16 cell hypoxic conditional medium.

In our previous study, we had identified an eIF3i mutant zebrafish harboring retrovirus insertion in the second exon of eIF3i which caused a premature stop codon in eIF3i open reading frame. We found that embryonic angiogenesis is disrupted in homozygous eIF3i mutant embryos, which were demonstrated to be partially caused by reduced VEGFa protein production in mutant embryos [15]. Ectopic overexpression of VEGFa in eIF3i mutant zebrafish rescued sprouting defects of the tip cells at 24 hours post fertilization (hpf), but did not rescue their vascularization. This result led us to ask whether eIF3i also functions in endothelial cells themselves. To test this hypothesis, we first determined if eIF3i was expressed in endothelial cells. We performed whole mount embryo in situ hybridization experiments (WISH) with an eIF3i specific antisense RNA probe, and detected strong positive signals within endothelial cells of zebrafish embryos at 24 hpf (Figure 1A). However, in embryos older than 3 days, no strong signals was detected (data not shown), suggesting that eIF3i is highly expressed during early stage of embryonic angiogenesis.

Likewise, we also found eIF3i expression in human endothelial cells by qPCR with eIF3i specific primers and by immunofluorescence (IF) and western blot (WB) with an eIF3i specific antibody (Figure 1B-1D). The specificity of qPCR and WB results were further confirmed by two non-overlap eIF3i siRNAs, both of which greatly reduced the RNA and protein levels. Because tumor angiogenesis shares many common features with embryonic angiogenesis, we sought to test if the expression level of eIF3i is higher in endothelial cells received tumor induction than that in normal control cells. We treated HUVECs with cancer cell conditional medium derived from mouse melanoma B16 cells (B16-CM). We found that 3 days treatment of B16-CM slightly increased eIF3i protein expression to 114% compared with control basic medium (BM). Conditional medium derived from hypoxic B16 cells (B16-HCM) further increased eIF3i protein expression to 167% (Figure 1E). To test if the major pro-angiogenesis molecule, VEGF-A, can promote endothelial eIF3i expression, we treated HUVEC with conditional medium containing VEGF-A. As shown in Figure 1E, this treatment also significantly increased eIF3i protein expression. Taken together, these results demonstrated that eIF3i expression is induced in endothelial cells by signals promoting embryonic and tumor angiogenesis.

### eIF3i is critical for endothelial cell proliferation and migration

**Figure 2:**
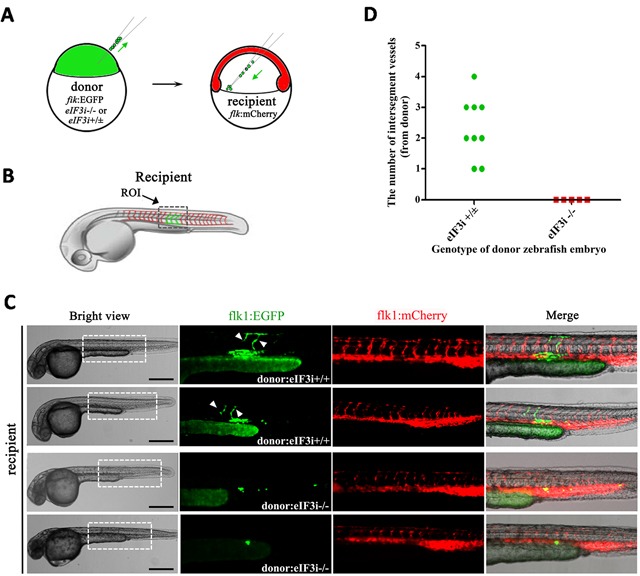
eIF3i acts an autonomous function in zebrafish vascular endothelial cells **A**. Schematic diagram of zebrafish embryos cell transplantation. The donors were wildtype or eIF3i−/−; flk1:GFP transgenic zebrafish embryos. The recipients were wildtype flk1:mCherry zebrafish embryos. The green arrows point to the operation direction. **B**. Diagram showing the region of interest (ROI) that was used for quantification of angiogenesis. **C**. Representatives of host embryos receiving either wildtype or eIF3i−/− donor cells at 48 hpf. The white arrowheads point to intersegment vessels derived from donor cells. Scale bars, 500μm. **D**. Quantification of donor derived intersegment vessels.

Next we sought to investigate the function of eIF3i in endothelial cells by analyzing the behaviors of endothelial cells lacking eIF3i activity with a cell transplantation assay. We crossed eIF3i mutant zebrafishes with the flk1:EGFP transgenic fishes expressing EGFP in their endothelial cells to obtain eIF3i-/+; flk1:EGFP lines. Then we transplanted blastula cells of donor embryos from an intercross of eIF3i-/+; flk1:EGFP parents into the ventral marginal region of the 6 hpf eIF3i wildtype host embryos harboring flk1:mcherry transgene. Schematic diagrams of transplantation were shown in Figure 2A and 2B. Cells in the lateral marginal region at this stage tend to differentiate into blood and vascular cells at later stage [16, 17]. The genotypes of the donor embryos were evaluated by their morphologies and were further confirmed by the presence of retrovirus sequence. At 48 hpf (Figure 2C and 2D), we found that endothelial cells from eIF3i wildtype or heterozygous embryos contributed to both axis and intersegment vessels and showed an undistinguished morphology to their neighboring host endothelial cells. However, endothelial cells derived from eIF3i homozygous mutant embryos showed an obviously distinguishable round shape. These cells never migrated dorsally to form intersegment vessels. By contrast, their neighboring host endothelial cells migrated normally and formed both axis and intersegment vessels, indicating that transplantation of mutant eIF3i embryonic cells had not interrupted the inductive environment for endothelial cells to migrate. This observation suggested that eIF3i functioned in an autonomous manner in endothelial cells.

**Figure 3:**
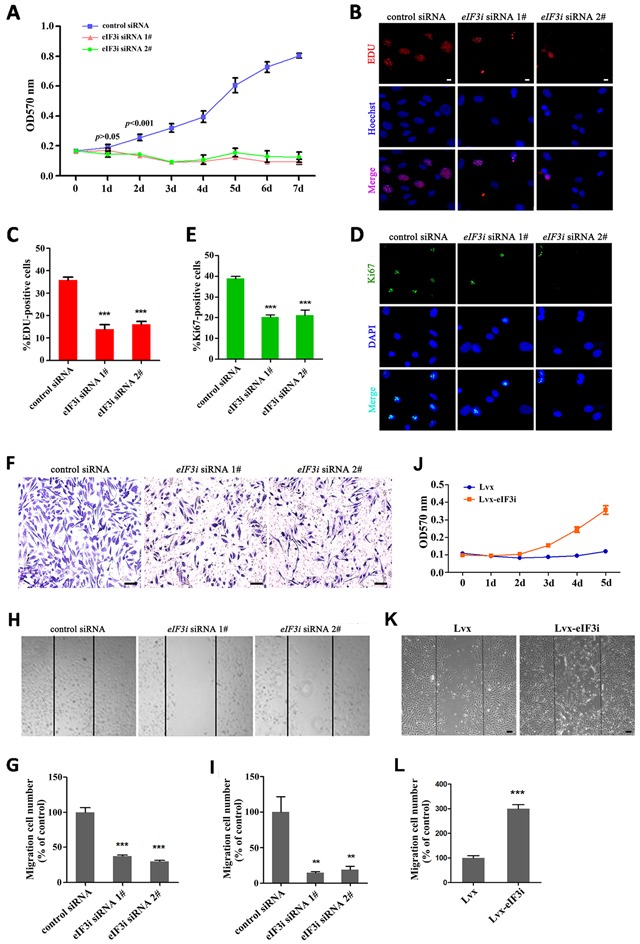
eIF3i promotes vascular endothelial cells proliferation and migration **A**. eIF3i siRNAs suppressed the HUVEC viability. MTT, 3-(4,5-dimethyl-2-thiazolyl)- 2,5-diphenyl-2-H-tetrazolium bromide (n=2). **B**. EDU assays in control siRNA- and eIF3i siRNAs-transfected HUVECs. Scale bars, 10μm. **C**. The statistics of EDU positive cells. (n=3). ***p< 0.001. **D**. Ki-67 immunofluorescent staining in control siRNA- and eIF3i siRNAs-transfected HUVECs. Scale bars, 10μm. **E**. The statistics of Ki-67 positive cells. (n=3). ***p<0.001. **F**. Transwell assay of control siRNA- and eIF3i siRNAs-transfected HUVECs. Scale bars, 100μm. **G**. The statistics of invaded cells in transwell assay. **H**. eIF3i siRNAs inhibited HUVECs migration in heal wound assay. Scale bars, 100μm. **I**. The statistics of cell migration. (n=3). **p<0.01. **J**. eIF3i overexpression increased the HUVEC viability. (n=3). **K**. eIF3i overexpression increased HUVECs migration in heal wound assay. Scale bars, 100μm. **L**. The statistics of cell migration. (n=3). ***p< 0.001.

To gain further insight into the function of eIF3i in endothelial cells, we used human HUVEC as an *in vitro* model. We transfected 2 non-overlap siRNAs against eIF3i into HUVECs, both of which significantly reduced proliferation of HUVECs as determined by MTT assay (Figure 3A). In consistent with this observation, an analysis of EDU incorporation revealed that both eIF3i siRNAs decreased the percentage of EDU positive cells to about 15%. By contrast, about 35% cells were EDU positive in HUVEC cells transfected with control siRNA (Figure 3B and 3C). This result was further confirmed by Ki67 staining, a cell proliferation marker, in which eIF3i siRNA also reduced the percentage of proliferating cells was observed in HUVECs and HMEC-1 (Figure 3D, 3E and Supplementary Figure 1A, 1B). Additionally, overexpression of eIF3i could increase the ability of proliferation of HUVECs (Figure 3J).

Next, we tested the effect of silencing eIF3i expression on cell migration in HUVEC cells using a transwell migration assay. Equal amount of HUVECs transfected with either control- or eIF3i-siRNAs were loaded onto the polycarbonate filters, and cells that migrated to the lower surface of the filter were counted under a light microscope. As shown in Figure 3F and 3G, the number of invaded cells decreased significantly in eIF3i knockdown HUVECs, compared with that of control cells. We further measured the effect of eIF3i knockdown on cell migration using a wound healing assay in HUVECs and HMEC-1. A fixed-width scratch was created in a cell monolayer 48 hours post-transfection with control- or eIF3i-siRNAs, and the progress of the migrating front was monitored under microscope. In comparison to cells transfected with scrambled siRNA, treatment of both eIF3i siRNAs significantly decreased cell migration (Figure 3H, 3I and Supplementary Figure 1C, 1D). In line with this observation, overexpression of eIF3i by lentivirus transfection in HUVECs significantly increased the migration (Figure 3K, 3L). Taken together, those data suggested that eIF3i is critical for endothelial cell proliferation and migration.

### eIF3i promotes VEGFR/ERK signaling

**Figure 4:**
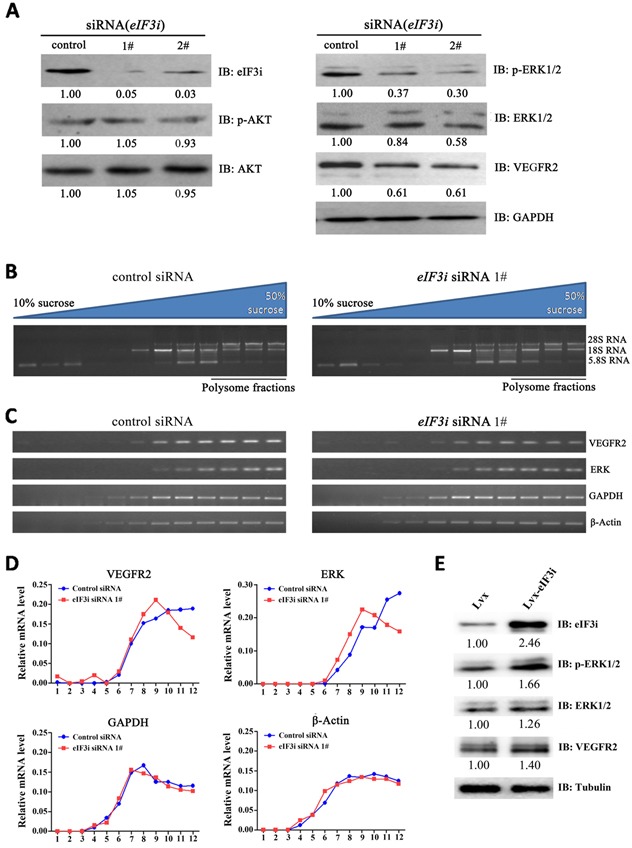
eIF3i selectively promotes VEGFR2 and ERK translation **A**. eIF3i siRNAs inhibited ERK/VEGFR2 signaling pathway in HUVECs. GAPDH was used as a loading control. Fold changes were compared with control-siRNA and presented below each blot. **B**. Polysomes from control siRNA- and eIF3i siRNA-transfected HUVECs were fractionated with sucrose gradients. Upper panel showed the schematic diagram of the 10-50% sucrose gradients, and lower panel showed the electrophoresis results of RNAs extracted from the fractions. **C**. Levels of PCR products of indicated genes were analyzed with agarose gel electrophoresis. **D**. VEGFR2, ERK, β-actin and GAPDH PCR products were quantified with ImageJ software. **E**. Overexpression of eIF3i increased the ERK and VEGFR2 protein level in HUVECs. Tubulin was used as a loading control. Fold changes were compared with Lvx and presented below each blot.

It has been reported that AKT phosphorylation is largely activated by eIF3i in HCC cell lines [14] and AKT phosphorylation enhance endothelial cell activation, therefore it is reasonable to ask whether eIF3i-AKT mechanism is also functioned in endothelial cell. We performed western-blot analysis to evaluate the phosphorylation level of AKT in HUVECs transfected with either control or eIF3i siRNAs. But the result showed that silence of eIF3i activity did not obviously alter the phosphorylation level of AKT (Figure 4A), suggesting that the eIF3i-AKT mechanism did not function in endothelial cells. Then we tested the phosphorylation levels of another key regulator in endothelial cell activation, ERK. The western blot analysis revealed that transfection of eIF3i siRNAs did downregulate p-ERK level and total ERK protein level (Figure 4A). We also found a significant reduction in the protein level of VEGFR2, an upstream regulator of ERK, in eIF3i siRNA treated cells (Figure 4A). These results were further confirmed with specific ELISA assay (Supplementary Figure 2). In addition, to exclude cell specific effect, we also tested this phenomenon with another endothelial cell line, HMEC-1, and obtained similar observations (Supplementary Figure 1E). Consistently, overexpression of eIF3i increased the expression of p-ERK, ERK and VEGFR2 in HUVECs (Figure 4E).

Considering that eIF3i is a key translational factor and that eIF3i has been found to contribute selective translational control, these results suggested eIF3i is required for the VEGFR and ERK translation in endothelial cells. To test this hypothesis, we performed polysome analysis. Cell extracts from control and eIF3i siRNA treated HUVECs were fractionated using 10-50% sucrose gradient to isolate polysome-bound mRNA (Figure 4B). Then the levels of VEGFR2, ERK, β-actin and GAPDH mRNAs were analyzed by RT-PCR. As shown in Figure 4C and 4D, eIF3i siRNA led to a shift of VEGFR2 and ERK mRNAs from polysome fractions toward monosome fraction, but had no effect on distributions of β-actin and GAPDH mRNAs, suggesting that eIF3i depletion selectively reduced translation levels of VEGFR2 and ERK mRNAs. Taken together, these data suggested that eIF3i promoted VEGFR/ERK signaling via selective translational control of VEGFR2 and ERK mRNA in HUVECs.

### eIF3i is critical for endothelial cell to respond to tumor derived induction signals

**Figure 5:**
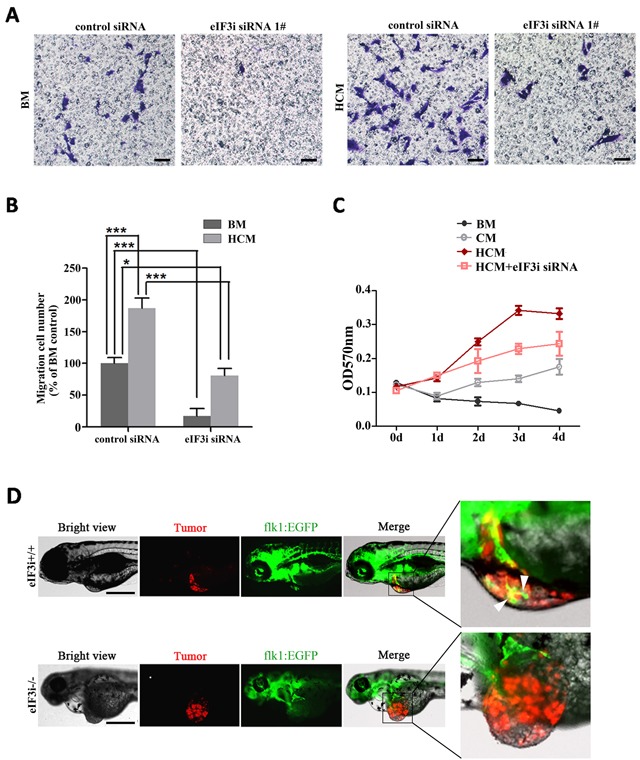
eIF3i is required for vascular endothelial cells to respond to tumor signals **A**. Knockdown of eIF3i suppressed tumor induced HUVECs migration. BM, basic medium. HCM, hypoxic conditional medium. Scale bars, 100μm. **B**. The statistics of invaded cell. BM/control siRNA (n=4), BM/eIF3i siRNA 1# (n=2), HCM/control siRNA (n=4), HCM/eIF3i siRNA 1# (n=4). *p<0.05, ***p< 0.001. **C**. Knockdown of eIF3i inhibited tumor induced HUVECs proliferation. CM, conditional medium. (n=3). **D**. eIF3i loss-of-function inhibits tumorigenic angiogenesis in zebrafish B16-RFP melanoma cells xenografts. The red channel represents tumor, and the green channel represents the blood vessels. The white arrowheads point to endothelial cell migrated into tumor mass. Scale bars, 500μm.

Next, we sought to study the effects of eIF3i loss-of-function on tumor induced angiogenesis. We performed a transwell assay to analyze the activation of endothelial cells by tumor derived signals. As shown in Figure 5A and 5B, B16-HCM increased the migration of HUVECs as compared to basic medium. However, this effect was significantly reduced by eIF3i knockdown. Meanwhile, we tested the effects of B16-CM and B16-HCM on the endothelial cell proliferation. As shown in the Figure 5C, HCM could boost the proliferation of HUVECs, and the effects were partially inhibited by eIF3i knockdown.

We also performed an *in vivo* angiogenesis assay by using transgenic zebrafish embryos. We transplanted B16-RFP cells into the abdominal perivitelline space of 2 days old wildtype or eIF3i mutant zebrafish embryos which also harbor FLK1-EGFP transgene and recorded the induction of endothelial cell migration toward tumor mass in the following 2 days. Similar to previous description [18], we observed obvious migrations of endothelial cell toward B16 tumor mass as early as 1 day post transplantation, and the number and degree of induced endothelial cells increased with the size of tumor mass (data not shown). At 2 days post transplantation, in all the B16 neoplasia, the endothelial cells had penetrated into tumor mass and preferred to form endothelial loops, and finally, these loops assembled into a complex vascular network in eIF3i wildtype or heterozygous embryos (Figure 5D). However, in eIF3i mutant embryos, no migrations of endothelial cell toward tumor mass were observed throughout this time window (Figure 5D). Therefore, our data suggested that eIF3i is critical for endothelial cell to respond to tumor derived signals.

### Inhibition of eif3i reduced tumor metastasis *in vivo*

**Figure 6:**
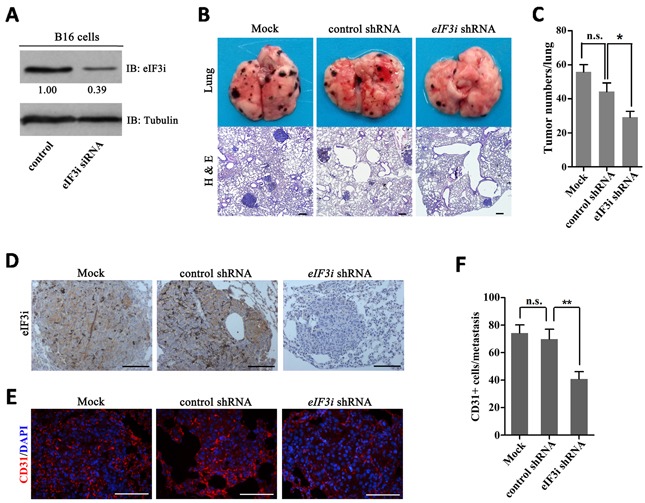
Inhibition of eIF3i reduced mouse melanoma metastasis **A**. Mouse eIF3i siRNA could effectively interfere the expression of eIF3i in B16 cells. Tubulin was used as a loading control. Fold changes were compared with control and presented below each blot. **B** and **C**. eIF3i shRNA significantly inhibit growth and metastasis of tumor in mouse pulmonary metastasis model (n=5). H & E, hematoxylin-eosin staining. n.s., not significant. *p<0.05. **D**. eIF3i shRNA reduced the expression of eIF3i in mouse melanoma metastasis. Mock (n=6), control shRNA (n=4), eIF3i shRNA (n=3). Scale bars, 100μm. **E**. eIF3i shRNA reduced the level of CD31 in mouse melanoma metastasis. Mock (n=17), control shRNA (n=15), eIF3i shRNA (n=15). Scale bars, 100μm. **F**. The statistics of CD31+ cell numbers in each metastasis section. n.s., not significant. **p<0.01.

In addition to supplying blood, endothelial cells also secrete a panel of growth factors to support tumor cell survival and proliferation. The fact that eIF3i functions in both tumor cells [10, 14, 15, 19]] and endothelial cell during tumor angiogenesis suggested it can be an attractive therapeutic target. To test this hypothesis, firstly, we designed a mouse eIF3i siRNA to transfect the B16 cells, finding that the siRNA could efficiently inhibit the production of eIF3i protein (Figure 6A). Then, we constructed an shRNA vector with a same target to treat melanoma metastasis in mouse model. As shown in Figure 6B and 6C, eIF3i shRNA treatment efficiently inhibited metastases of mouse melanoma. Upon eif3i shRNA treatment, eIF3i proteins, as well as CD31 proteins, were significantly reduced in melanoma metastasis (Figure 6D-6F). Taken together, our data suggested that eIF3i could be an effective target for anticancer therapy.

## DISCUSSION

Accumulating evidence shows that translational control plays fundamental roles for cells to respond to intra- and inter-cellular signals by reprogramming their proteomes. Rather than globe, the translational control of a subgroup of mRNAs tends to be selective when cells are exposed to specific environments. eIF3 complex is critical for the control of selective translation. This complex is the largest and most complicated translation initiation factor comprising 13 non-identical protein subunits in mammalian cells [9, 20]. Previously, we and others had shown that eIF3 subunits were extensively involved in selectively translational control [15, 21–23]. Recently, several eIF3 subunits, such as 3a, 3b, 3c, 3h, 3i and 3m, were shown to be induced by aberrant signals during carcinogenesis and metastasis, leading to up-regulation in the translation of specific mRNAs, whose products were involved in the control of a variety of cell behaviors, including cell growth, angiogenesis, and cell migration [24].

**Figure 7:**
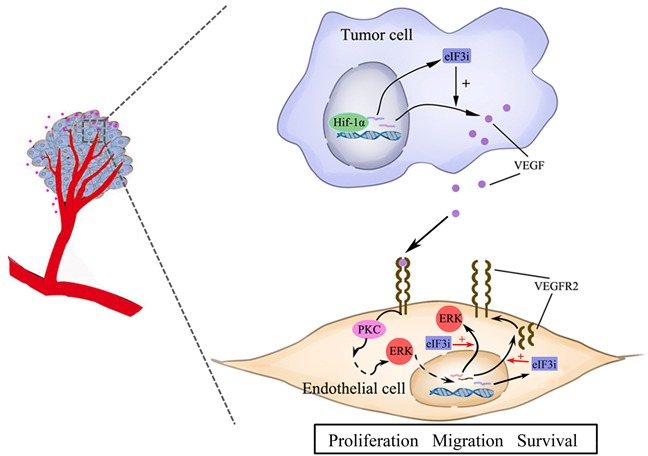
The summary of eIF3i function in tumor and endothelial cells

In this work, we found that the expression of eIF3i in endothelial cells is significantly up-regulated during embryonic and tumor angiogenesis. During early embryonic development, eIF3i is highly expressed in the vascular endothelial cells. However, its expression withered with the development and maturation of embryonic vascular system. And in cultured HUVECs, eIF3i is significantly induced by hypoxic-tumor-derived signals. These observations suggested eIF3i expression is dynamically controlled by extracellular signals that guide endothelial cells to proliferation and migration. Upon activation, eIF3i promote cell growth and migration of endothelial cells by selectively up-regulating ERK and VEGFR2 translation (Figure 7). Knockdown of eIF3i greatly attenuated the response of endothelial cells to the induction signals from tumor cells. Without eIF3i activity, endothelial cells can never migrate toward tumor and tumor angiogenesis was blocked. Thus, these findings suggested an important interaction between endothelial cells and cancer cells, in which eIF3i is a key regulator linking cancer signals to endothelial cell response. Given that eIF3i is also critical for the proliferation and survival of cancer cells, and that eIF3i is required for the translational activation of VEGF-A, these findings suggested eIF3i could be an important target for cancer therapy.

## MATERIALS AND METHODS

### Zebrafish husbandry

Zebrafish were bred and maintained normally (28°C; pH 7.2-7.4). Genotype of eIF3i mutant zebrafish was determined by PCR as previous described [15]. The treatment and handing of zebrafish have been approved by Sichuan Animal Care and Use Committee and performed under relevant guidelines. The Permit Number is SYXK (Chuan) 2008-119.

### Cell transplantation in zebrafish

For tumor cells transplantation, 50-100 cells were injected into perivitelline space of zebrafish embryos by using an electronically regulated air-pressure micro-injector [18] (Harvard Apparatus, NY, PL1-90). Transplantation of blastula cells into shield-stage embryos was performed as described previously [25]. About 30 cells were transplanted into lateral marginal zones of host embryos with an oil-pressure micro-injector (Eppendorf).

### In situ hybridization

eIF3i probe was synthesized with a digoxigenin labeling kit (Roche Applied Science). Whole-mount in situ hybridizations were performed as previously described [26].

### Cell culture and transfection

B16-F10 was obtained from the American Type Culture collection (ATCC, Manassas, VA), and cultured in RPMI-1640 supplemented with 10% fetal bovine serum (FBS). B16-RFP cells were infected with LV-RFP, and sorted by flow cytometer. HUVECs were isolated from human umbilical cord veins as previously described, and cultured in EBM-2 medium [27]. siRNAs were transfected with Lipofectamine RNAiMAX Reagent (Life Technologies) according to manufacturer's instructions. Following eIF3i siRNAs were used: human eIF3i siRNA 1# (5′-GGTCCATTACGCAGATTAA-3′), human eIF3i siRNA 2# (5′-GACAGAACGTCCTGTCAAC-3′) and mouse eIF3i siRNA (5′-GGTCCATCACGCAGA TCAA-3′).

### Conditioned medium

To prepare B16 hypoxic conditioned medium (HCM), B16-F10 cells placed into a mimic hypoxic incubator with 5% CO2, 1% O2 and 94% N2 for 24h [28]. The B16 non-hypoxic conditional medium (CM) was prepared under 21% O2. The conditioned medium containing overpressed VEGF (CM-VEGF) was prepared by VEGF plasmid transfection.

### Transwell assay

After 48 hr of transfection with control siRNA and eIF3i siRNAs, the HUVECs were harvested, and resuspended with EGM-2 basic medium at a concentration of 5×105 cells/ml. Then 100 μl cell suspension was added into the top chambers and the bottom chambers were filled with EGM-2 completely medium. Cells were allowed to migrate for 24 hr. Nonmigrated cells were erased with a cotton swab, and migrated cells were fixed with methanol and stained with crystal violet.

### Wound healing

After 48 hr of transfection with control siRNA and eIF3i siRNAs, the monolayer HUVECs were wounded by scratching with a pipette tip and washed three times with PBS. Cells were allowed to migrate for 24 hr. Images were taken by a Zeiss digital camera, and the migration was quantified with ImageJ software.

### Western blot

Cells were lysed by RIPA buffer supplemented with cocktail proteinase inhibitor. Then the samples were separated by SDS-PAGE gel electrophoresis, and electroblotted onto polyvinylidene fluoride membrane. After blocked with 5% BSA or nonfat milk, membranes were incubated overnight at 4°C with primary antibodies as indicated. Blots were developed with secondary horseradish peroxidase (HRP)-conjugated antibody, and signals were exposed onto x-ray films. The following antibodies were for Western blot: eIF3i(Abcam), phospho-AKT (p-AKT, Ser473; Cell Signaling technology), AKT (Cell signaling technology), phospho-ERK1/2 (p-ERK1/2, Thr202/Tyr204; Cell signaling technology), ERK1/2 (Cell signaling technology), VEGFR2 (Abcam), GAPDH (Tianjin sungene Biotech), Tubulin (Tianjin sungene Biotech).

### RT-PCR

Total RNA was extracted by using Trizol reagent (Life Technologies), and the cDNA was synthesized using Revert Aid First strand cDNA Synthesis Kit (Thermo). Real-time PCR was performed using the SsoAdvanced SYBR Green Supermix (Bio-Rad) on a CFX96 PCR System (Bio-Rad). Gene expression was normalized to GAPDH. Gene-specific primers were listed in Supplementary Table 1.

### MTT assay

HUVECs were treated with eIF3i siRNAs for different time intervals (0 hr, 24 hr, 48 hr, 72 hr, 96 hr, 120 hr). MTT was added at a concentration of 1mg/ml and spectrometric absorbance at 570 nm was measured with Multiscan MK3 ELISA reader (Thermo Scientific). Each assay was replicated 3 times.

### Immunofluorescence assay

The HUVECs were seeded onto coverslips in 24-well at a density of 30 000 cell/well. After transfection of eIF3i siRNAs for 72 hr, Ki-67 and EDU immunofluorescence were performed. For Ki-67 staining, the cells were fixed with prechilled acetone, blocked with goat serum, incubated with Ki-67 (Abcam) and stained with goat anti-rabbit IgG/FITC. EDU assay was completed according to manufacturer's specifications. The slides of cells were photographed with a Zeiss fluorescence micrograph.

### ELISA assay

Soluble VEGFR2 and ERK were quantified in HUVECs lysis using ELISA kits (Boster), following the manufacturer's instructions. HUVECs were collected at 48 hr after transfection and lysed by several cylcles of freezing and thawing. Proteins concentration were determined with NanoDrop 2000 (NanoDrop). Standard curve was made at the same time.

### Lentivirus package

The pLVX-eIF3i overexpression and pLVX plasmids were transfected into 293TL cells together with the packaging and envelop plasmids. Lentivirus was harvested 36-48 hours post transfection.

### Polysome analysis

Polysome analysis was performed as described previously [29]. Briefly, HUVECs were treated with 0.1mg/ml cycloheximide (CHX, Sigma) for 15 min and washed by prechilled PBS twice before collection. Then the cells were lysed with 10 mM HEPES (pH=7.8), 150 mM NaCl, 10 mM KCl, 1 mM EDTA (pH=8.0), 2 mM MgCl2, 10% glycerol, 0.5% Triton X-100, 1 mM DTT, 1 IU/μl RNase inhibitor. The lysate was centrifuged for 5 min at a speed of 13000 rpm, and the supernatant was loaded onto premade 10%-50% sucrose gradients. The tubes were centrifuged in a Beckman SW41Ti rotor at 35000 rpm for 3.5 hr at 4°C. Then the gradients were separated from top to bottom into 12 fractions, and performed by agarose gel electrophoresis and reverse transcription PCR analysis. Gene-specific primers were listed in Supplementary Table 1.

### Mouse tomor xenograft model

B16 tumors were established by tail vein injection of 1×104 B16 cells (in 0.1 ml serum free RPMI-1640). Then, these mice were randomly allocated into 3 groups (5 mice per group). Mice were treated with liposomal encapsulated plasmid DNA (5 μg per mice) in 100 μl 5% glucose solution 3 days after inoculation and the subsequent treatments were performed every two days. After 5 times of treatment, mice were anesthetized and sacrificed. The lungs of mice were removed, and the tumors within lung surfaces were counted.

### Statistical analysis

GraphPad Prism 5 was used for statistical analysis. Data were expressed as mean ± SEM, and analyzed by Students’ t-test. Differences were considered significant when p<0.05.

## SUPPLEMENTARY FIGURES AND TABLES


