# Supplementary material for: eIF3i activity is critical for endothelial cells in tumor induced angiogenesis through regulating VEGFR and ERK translation
Source: Oncotarget. 2017 Feb 11;8(12):19968–79. doi: 10.18632/oncotarget.15274 (PMC5386737; doi:10.18632/oncotarget.15274)
Supplement: Supplementary file 1 [file oncotarget-08-19968-s001.pdf]

eIF3i activity is critical for endothelial cells in tumor induced angiogenesis through regulating VEGFR and ERK translation

SUPPLEMENTARY FIGURES AND TABLE

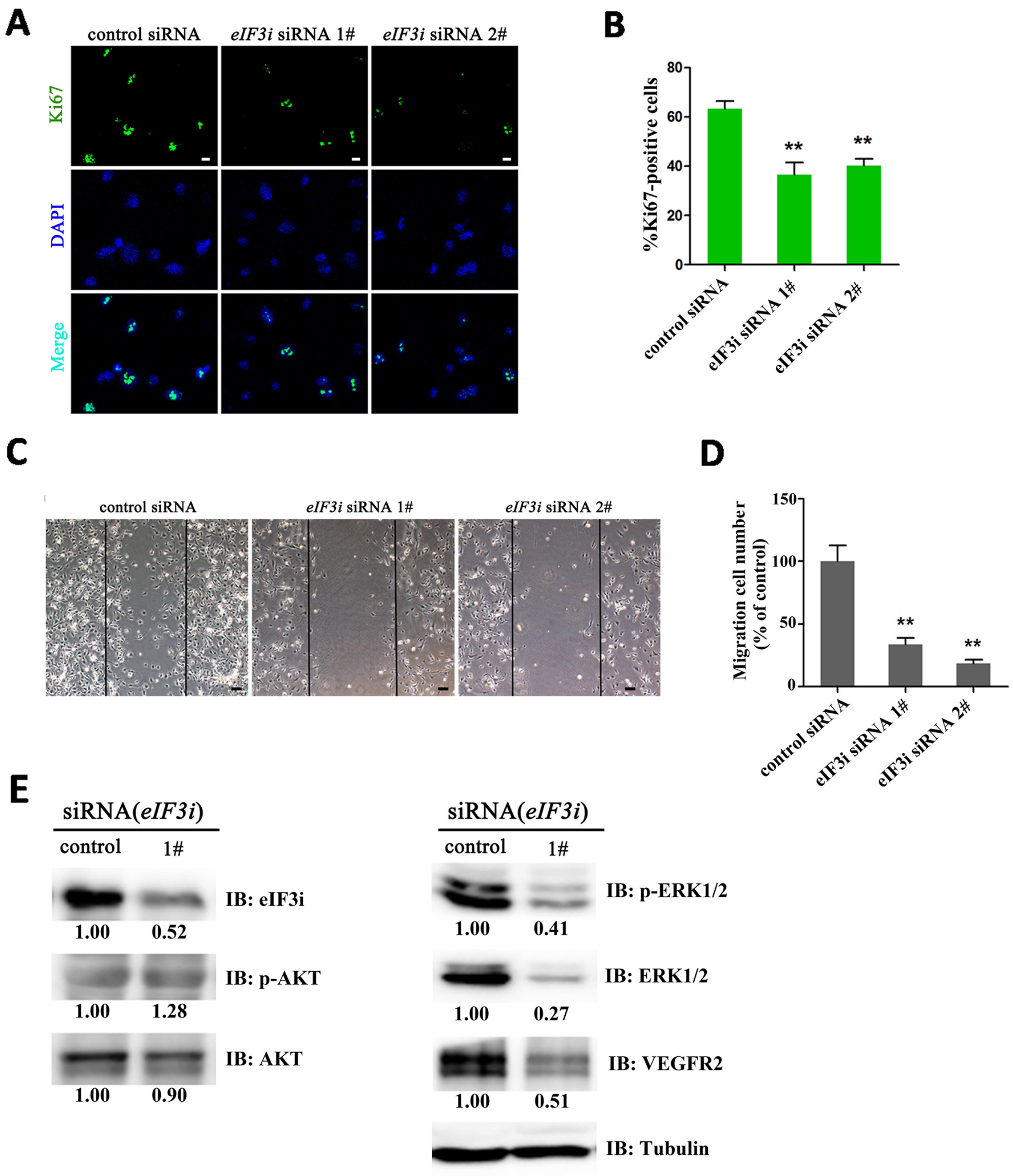

Supplementary Figure 1: eIF3i promotes HMEC-1 cells proliferation, migration and the expression of ERK and VEGFR2. **A.** Ki-67 immunofluorescent staining in control siRNA- and eIF3i siRNAs-transfected HUVECs. Knockdown of eIF3i in HMEC-1 significantly reduced the expression of Ki-67. Scale bars, 10μm. **B.** The statistics of Ki-67 positive cells. (n=3). **p<0.01. **C.** eIF3i siRNAs inhibited HMEC-1 migration in heal wound assay. 48 hr post siRNA transfection, HMEC-1 were wounded and allowed to migrate for 24 hr. Scale bars, 100μm. **D.** The statistics of cell migration. (n=3). **p< 0.01. **E.** eIF3i siRNAs inhibited ERK/VEGFR2 signaling pathway in HMEC-1. Tubulin was used as a loading control. Fold changes were compared with control-siRNA and presented below each blot.

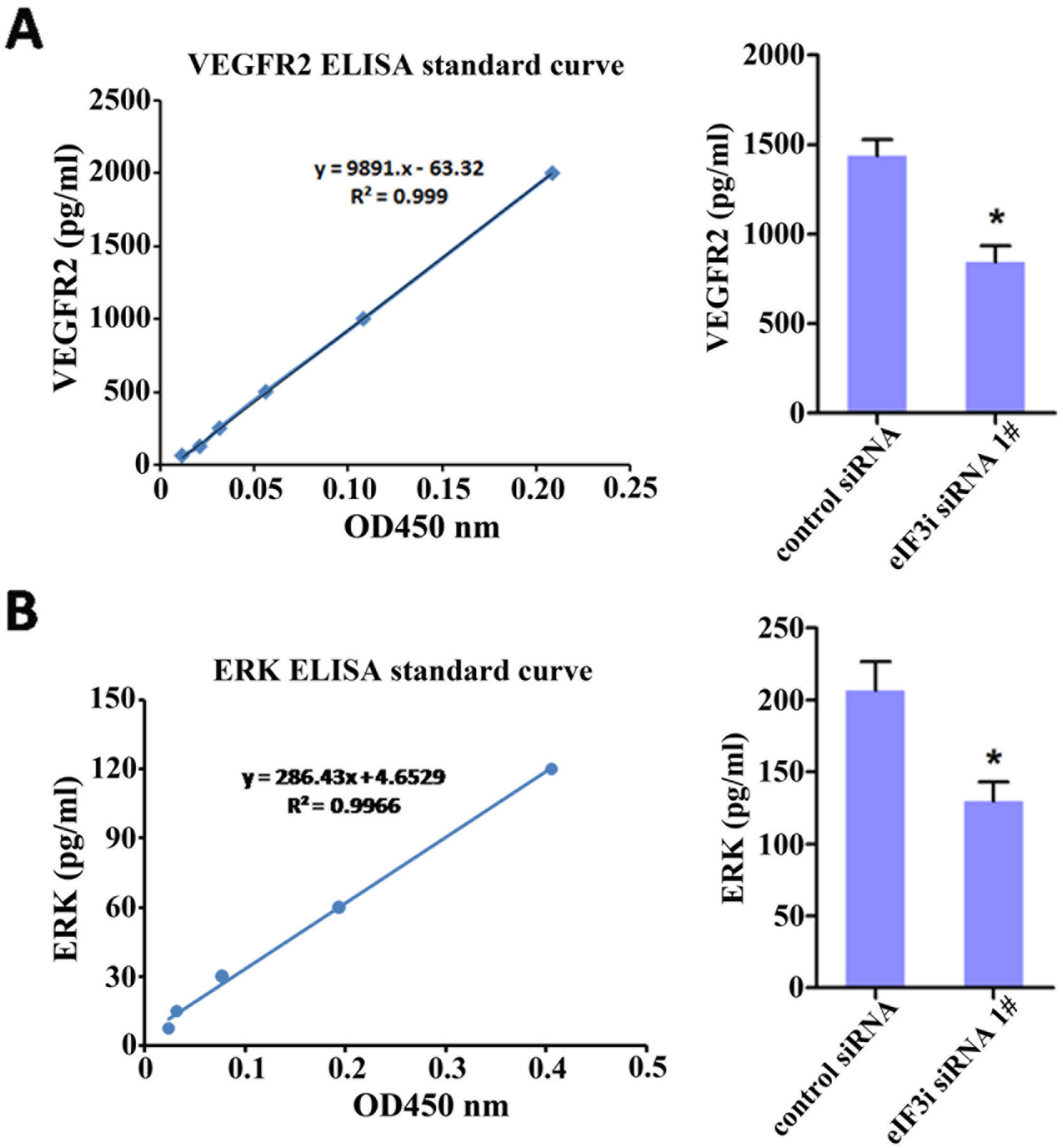

Supplementary Figure 2: eIF3i promotes the expression of ERK and VEGFR2 in HUVECs. A. Quantification the expression of VEGFR2 in HUVECs via ELIAS. (n=3). *$p<0.05$. **B.** Quantification the expression of ERK in HUVECs via ELIAS. (n=3). *$p<0.05$.

Supplementary Table 1: Primers for real-time qPCR

Gene	Forward primer (5'-3')	Reverse primer (5'-3')
eIF3i	CTGTGGCCAAGGACCCTATC	CCAGTGAGGACATGCTTGGT
VEGFR2	GGCCCAATAATCAGAGTGGCA	TGTCATTTCCGATCACTTTTGGA
ERK	TCCTGAACTTCTGCAACCCC	AGAACCAAATGTCCAAGAAATCCT
GAPDH	AGAAGGCTGGGGCTCATTTG	AGGGGCCATCCACAGTCTTC
β-Actin	AGCGAGCATCCCCCAAAGTT	GGGCACGAAGGCTCATCATT
